# Study of Inheritance and Linkage of Virulence Genes in a Selfing Population of a Pakistani Dominant Race of *Puccinia striiformis* f. sp. *tritici*

**DOI:** 10.3390/ijms21051685

**Published:** 2020-02-29

**Authors:** Sajid Mehmood, Marina Sajid, Syed Kamil Husnain, Jie Zhao, Lili Huang, Zhensheng Kang

**Affiliations:** 1 State Key Laboratory of Crop Stress Biology for Arid Areas, College of Plant Protection, Northwest A&F University, Yangling 712100, China; sajid.mehmood@nwsuaf.edu.cn (S.M.); kangzs@nwsuaf.edu.cn (Z.K.); 2 College of Food Science and Engineering, Northwest A&F University, Yangling 712100, China; msajid1118@nwsuaf.edu.cn; 3 Plant Pathology Section, Barani Agricultural Research Institute, Chakwal 48800, Punjab, Pakistan; kamiluaf@gmail.com

**Keywords:** wheat stripe rust, *Puccinia striiformis*, *Berberis*, virulence, *Yr* genes, resistance, genetic inheritance

## Abstract

Wheat stripe rust is a severe threat of almost all wheat-growing regions in the world. Being an obligate biotrophic fungus, *Puccinia striiformis* f. sp. *tritici* (*PST*) produces new virulent races that break the resistance of wheat varieties. In this study, 115 progeny isolates were generated through sexual reproduction on susceptible Himalayan *Berberis pseudumbellata* using a dominant Pakistani race (574232) of *PST*. The parental isolate and progeny isolates were characterized using 24 wheat *Yr* single-gene lines and ten simple sequence repeat (SSR) markers. From the one-hundred-and-fifteen progeny isolates, 25 virulence phenotypes (VPs) and 60 multilocus genotypes were identified. The parental and all progeny isolates were avirulent to *Yr5*, *Yr10*, *Yr15*, *Yr24*, *Yr32*, *Yr43*, *YrSp*, *YrTr1*, *YrExp2*, *Yr26*, and *YrTye* and virulent to *Yr1*, *Yr2*, *Yr6*, *Yr7*, *Yr8*, *Yr9*, *Yr17*, *Yr25*, *Yr27*, *Yr28*, *YrA*, *Yr44*, and *Yr3*. Based on the avirulence/virulence phenotypes, we found that VPs virulent to *Yr1*, *Yr2*, *Yr9*, *Yr17*, *Yr47*, and *YrA* were controlled by one dominant gene; those to *YrSp*, *YrTr1*, and *Yr10* by two dominant genes; and those to *YrExp2* by two complementary dominant genes. The results are useful in breeding stripe rust-resistant wheat varieties and understanding virulence diversity.

## 1. Introduction

Wheat stripe or yellow rust, caused by *Puccinia striiformis* Westend. f. sp. *tritici* Eriks (*PST*) [1], is one of the most devastating disease of wheat worldwide [2,3], bringing an annual loss of ~1 million tons of wheat yield. Wheat stripe rust has been reported in more than 60 countries [4]. In China, severe stripe rust epidemics occurred in 1950, 1964, 1990, and 2002 [5,6]. In recent years, 1.65 million hectares of the wheat crop were affected by this pathogen in 12 provinces in China [7]. Although disease varies in its frequency of occurrence and impact of damage, almost every country has this problem, for example, in Australia, New Zealand, India, Nepal, China, Pakistan, Yemen, Uzbekistan, Kenya, Ethiopia, the United Kingdom, Peru, Chile, Colombia, Ecuador, Mexico, and the US, it caused 5–10% yield losses. The disease can cause a 100% yield loss on highly susceptible wheat cultivars. In the US, in 2011, more than 90% of yield loss was recorded on a susceptible check in an experimental field near Pullman, Washington [8]. Virulence and molecular characterization of experimental isolates of the stripe rust pathogen (*Puccinia* spp.) indicated somatic recombination [9]. A large number of *PST* races are evolved due to the recombination of virulence genes through sexual reproduction, hybridization, or mutations resulting in breaking the resistance of the wheat cultivars [10,11]. However, only a few races become dominant due to high virulence spectra and aggressiveness towards resistant wheat cultivars [12]. For example, in China, the C*YR32* race was considered as the most virulent predominant race [13], being detected for the first time in Huangzhong, Qinghai Province in 1991, on wheat cultivar Red Abbondanza [14]. This race was found virulent to many wheat differentials having resistance genes, including Hybrid 46 (*Yr*4b) in China [15]. This race has spread rapidly in the wheat-growing areas in China, and 45.7% frequency was observed following large-scale epidemics in China [15,16].

It was not possible to understand the role of sexual reproduction in generating virulence variation until the identification of *Berberis* and *Mahonia* species as the aecial hosts of *PST* [17], which made it possible to study the genetics of virulence genes and mapping them. However, under natural conditions, *PST* infection of *Berberis* spp. has been reported only in China [18,19,20,21]. In the Pacific Northwest of the United States and southeastern Sweden, *PST* infection was not detected on barberry plants, but *P. graminis* was common on the aecial host plants in these regions [22,23]. Zhao et al. [24] suggested that a large number of races may evolve due to sexual reproduction on alternate hosts under natural conditions.

The first study of the genetics of *PST* virulence was conducted by Wang et al. [25], by selfing a US isolate of race *PST*-127 on *B. vulgaris* and generating 29 progeny isolates. They tested the isolates on *Yr* single-gene lines and found a parental isolate as homozygous for virulent loci to *Yr1*, *Yr2*, and *Yr9* and for avirulence loci to *Yr5*, *Yr15*, *Yr24*, *Yr32*, and *YrSp*, whereas its segregation was observed for virulence phenotypes (VPs) to *Yr6*, *Yr7*, *Yr8*, *Yr10*, *Yr17*, *Yr1*9, *Yr27*, *Yr32*, *Yr44*, *Yr*Exp1, *YrExp2*, *YrTr1*, and *Yr7*6 (*YrTye*) in different ratios. Rodriguez-Algaba et al. [26] inoculated previously identified barberry to study the genetics of avirulence/virulence and diversity within and among aecia of *PST* isolates produced on *B*. *vulgaris*. The genetic markers confirmed segregation and resulted that the progeny isolates were derived from the parental isolate through sexual reproduction.

Tian et al. [27] obtained 118 isolates by selfing a Chinese *PST* isolate (Pinglan 17-7) on *B*. *shensiana* and tested them on 24 *Yr* single-gene lines. They found 24 VPs and 82 MLGs using 13 polymorphic SSR markers. A preliminary linkage map was constructed with eight of 24 avirulence/virulence loci and 10 SSR markers. They found that a highly diversified genetic population of *PST* can be generated by selfing a single isolate on barberry. In another study [28] using the same method, 120 progeny isolates were generated. They tested the progeny isolates on 25 *Yr* single-gene lines and found 51 VPs and 55 MLGs using 11 polymorphic SSR markers. Another linkage map was constructed using four avirulence loci and 11 SSR markers.

Recently, Wang et al. [7] generated 127 progeny isolates by selfing a Chinese predominant race, *CYR32*, on *B*. *aggregate*. The progeny isolates and the parental isolate were tested on 25 wheat *Yr* single-gene lines for phenotypic diversity and 10 SSR markers for genotypic diversity. They found 27 virulence phenotypes (VPs) and 65 multilocus genotypes (MLGs). The parental isolate and all progeny isolates were avirulent to *Yr5*, *Yr8*, *Yr10*, *Yr15*, *Yr24*, *Yr26*, *Yr32*, and *YrTr1*, but virulent to *Yr1*, *Yr2*, *Yr3*, *Yr*4, *Yr25*, *Yr44*, and *Yr7*6. The heterozygous avirulence phenotypes by the parental isolate were found to nine *Yr* genes (*Yr6*, *Yr7*, *Yr9*, *Yr17*, *Yr27*, *Yr28*, *Yr32*, *YrA*, and *YrExp2*) and avirulence phenotype to *YrSp*. The segregation data showed that the VPs to *Yr7*, *Yr28*, *Yr32*, and *YrExp2* were controlled by a dominant gene; to *Yr6*, *Yr9*, and *YrA* (*Yr7*3, *Yr7*4) by two dominant genes; to *Yr17* and *Yr27* by one dominant and one recessive gene; and to *YrSp* by two complementary dominant genes, respectively. A linkage map of 10 virulence/avirulence genes was constructed using 10 SSR markers. 

Since 1948, in Pakistan, 13 epidemics of wheat stripe rust have been reported [29]. Outbreak on Inqilab-91 (*Yr27*) wheat variety during 2003 to 2004 was the most destructive, which affected 80% of the wheat-growing area in Pakistan [30]. In the past, a 10.1% (0.83 million tons) yield loss caused US$86 million dollars in losses during 1977 to 1978 [31], and US$8 million dollars in losses [32] in Baluchistan have been reported. During 1995, rust epidemics on Pak 81 and Pirsabak 85 were reported [33]. Hussain et al. [34] reported a loss of 2 billion Pakistani rupees due to this disease between 1997 and 1998. The frequency of the disease was high in the northern areas and less in the central and western highlands of the country. In the southern parts of the country, the disease occurred in the past due to the dry and hot environment, but recently, wheat is affected by *PST* in the southern parts of Punjab and Sindh province [35]. Pakistan produced 25,482 thousand tons of wheat from a total cultivated area of 9260 thousand hectares during 2015–16 (http://www.finance.gov.pk/survey/chapters_16/02_Agriculture.pdf). Approximately 70% (5.8 million ha) of the wheat-growing area in Pakistan is prone to *PST* [36]. The Punjab province covers an area of ~20.5 thousand km², and it shares 75% of the total cultivated area in Pakistan, which is more than 85% of the whole wheat production in the country. Although wheat stripe rust is a significant foliar disease and causes severe losses every year in Pakistan, work on the virulence of the pathogen is minimal [37,38,39]. Virulence information on *PST* populations is essential for the implementation of resistance genes in the wheat varieties to overcome the disease. Nonetheless, after a few years of deployment of resistant cultivars, new races of the rust pathogen emerge and cause severe stripe rust epidemics, resulting in failure of resistant genes. For example, in Pakistan and India, *Yr27* had been widely used in wheat cultivars and became susceptible after the emergence of new virulent races from 2002 to 2004 [39,40]. We used the most dominant stripe rust race 574232, having avirulence/virulence formula *Yr5*, *Yr10*, *Yr15*, *Yr*41, *Yr32*, *Yr*46, *YrTr1*, *YrTye*/*Yr1*, *Yr6*, *Yr7*, *Yr8*, *Yr9*, *Yr17*, *Yr*42, *Yr44*, and *Yr*45 [30], to generate a sexual population using artificial inoculation under greenhouse conditions. 

In this study, we analyzed the genetics of avirulence/virulence genes of a progeny population of one hundred and fifteen single urediniospores obtained by selfing a Pakistani dominant race of *PST* on a highly susceptible *Berberis* species (*B. pseudumbellata*) collected from the Himalayan region in Pakistan. The main objectives of this study were (i) to test the virulence diversity in the selfing population and (ii) to study inheritance and linkage of virulence genes using simple sequence repeat (SSR) markers.

## 2. Results

### 2.1. Virulence Phenotypes

**Figure 1:**
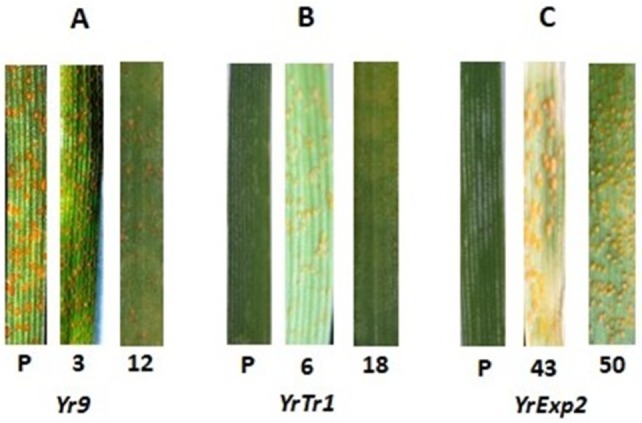
Infection types (ITs) scale bar: 0–9 produced by the parental isolate (P), [Pak-1-(A)-9] of a Pakistani dominant race (574232) of *Puccinia striiformis* f. sp. *tritici* (*PST*) selfed on *Berberis pseudumbellata*, and its progeny isolates (3, 12, 6, 18, 43, and 50) on wheat *Yr* single-gene lines (**A**) *Yr9*; (**B**) *YrTr1*, and (**C**) *YrExp2*.

A total of one-hundred-and-fifteen single-spore isolates were generated through sexual reproduction by artificially inoculating the Himalayan barberry (*B*. *pseudumbellata*) with a Pakistani dominant race (574232) of *PST*. The progeny isolates were homozygous avirulent to eight single-gene lines (*Yr5*, *Yr10*, *Yr15*, *Yr24*, *Yr32*, *Yr43*, *YrTye*, and *Yr26*), homozygous virulent to six single-gene lines (*Yr6*, *Yr7*, *Yr8*, *Yr25*, *Yr44*, and *Yr3*), and segregated on 10 wheat *Yr* single-gene lines (*Yr1*, *Yr2*, *Yr9*, *Yr17*, *Yr27*, *Yr47*, *YrA*, *YrSp*, *YrTr1*, and *YrExp2*). These results show that the parental Pakistani isolate was homozygous avirulent for the first group of eight avirulence loci, homozygous virulent for the second group of six virulence loci, and heterozygous for the third group of ten loci. A total of 25 VPs were identified. The VP1 was the largest group, comprised of 42 isolates having the same virulence pattern as the parental isolate virulent to six and avirulent to four of the 10 wheat *Yr* single-gene lines, respectively. Compared to the parental isolate, VP2, the second largest group, consisting of 25 VP, changed from virulence to avirulence (*Yr2*) and from avirulence to virulence (*YrTr1*). Eleven VPs (VP8 to VP18), comprised of 24 isolates, showed an increased number of virulences, whereas the remaining seven VPs (VP19 to VP25) showed a decreased number of virulences. These results (Table 1) show that the single isolate of a dominant Pakistani race of *PST* sexually produced a large number of races with more or less virulence as compared to the parental isolate, as shown in Figure 1.

### 2.2. Inheritance of Virulence

In this study, different segregation ratios were obtained for the heterozygous avirulence/virulence loci. The avirulence phenotypes to *YrSp*, *YrTr1*, and *Yr47* were controlled by a single recessive gene or the VPs were controlled by a single dominant gene, indicated by 1:3 avirulent to virulent ratio. The avirulence phenotypes to *Yr1*, *Yr2*, *Yr9*, *Yr17*, *Yr27*, and *YrA* were controlled by two complementary recessive genes, indicated by 1:15 avirulent to virulent ratio. For *YrExp2*, the avirulence to virulence ratio of 7:9 fits best, indicating that the parental isolate had two independent recessive genes for virulence phenotype (Table 2).

### 2.3. Simple Sequence Repeat (SSR) Markers and Genotyping

A set of 141 SSR markers specific for *PST* were screened and 10 produced co-dominant amplicons in the parental isolate and segregated in the progeny population. The selected 10 SSR markers and DNA samples of one-hundred-and-fifteen progeny isolates were genotyped and different amplicons were produced by these markers. The heterozygous and homozygous amplicons produced by SUNI*Pst*11–10 in the parental isolate, and three progeny isolates are shown in Supplementary Figure S1. The correlation coefficient between avirulence phenotypes and SSR genotypes was found very low (*R*^2^ = 0.06), as it was expected in this study. A total of 60 multilocus genotypes (MLGs) were identified from one-hundred-and-fifteen progeny isolates (Supplementary Table S4). The first multilocus genotype (MLG1) was identified from three progeny isolates, and it was the same as the parental isolate showing the heterozygosity at all of the 10 SSR marker loci. The remaining 59 MLGs were different from the parental isolate by different numbers of 1–9 of the homozygous marker loci. The calculated frequency distribution of the number of marker loci becoming homozygous was close to the normal distribution based on progeny isolates and MLGs. This shows that the one-hundred-and-fifteen progeny isolates were randomly generated, and the selected SSR markers were suitable to construct the linkage map.

### 2.4. Linkage Map Construction of Virulence Genes

**Figure 2:**
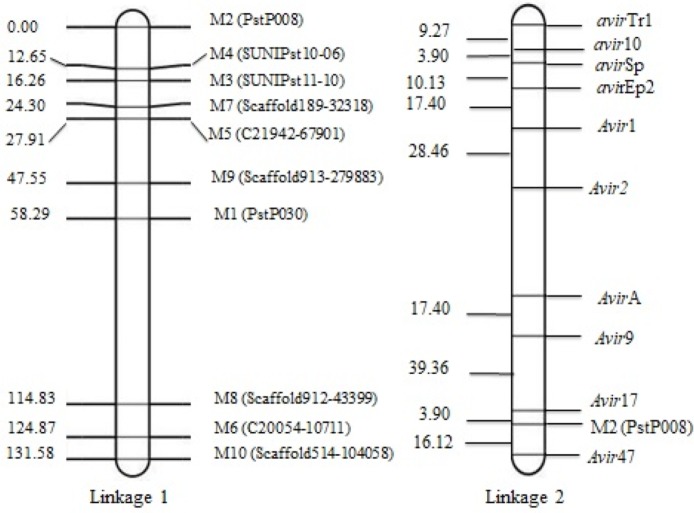
Genetic maps constructed with QTL Ici Mapping; linkage 1 consists of 9 SSR markers and linkage 2 consists of 1 SSR marker and 10 avirulence/virulence genotypes.

Using the 10 VPs and the 10 segregating SSR markers, a map consisting of two linkages was constructed. Linkage 1 consisted of nine SSR markers and linkage 2 consisted of one SSR marker and 10 avirulence/virulence genes. The genetic distance between two neighboring virulence loci ranged from 3.90 cM between *avir10* and *avirSp*, and 39.36 cM between *Avir9* and *Avir17*, respectively. The marker M2 (*Pst*P008) linked very close to *Avir17* clustering at one end of the chromosome. Linkages 1–9 of SSR markers spanning 131.58 cM show genetic distances ranged from 3.59 cM between M5 (C21942-67901) and M7 (Scaffold189-32318) and 19.64 cM between M9 (Scaffold913-279883) and M5 (C21942-67901), respectively (Figure 2).

## 3. Discussion

In this study, a sexual population of one-hundred-and-fifteen isolates was generated by selfing an isolate of a Pakistani dominant race (574232) of *PST* on the Himalayan barberry (*B*. *pseudumbellata*). By testing the parental and progeny isolates on 24 wheat *Yr* single-gene lines, the parental isolate was found to be homozygous for its avirulence phenotypes to *Yr5*, *Yr10*, *Yr15*, *Yr24*, *Yr32*, *YrSp*, *YrTr1*, *YrTye*, *YrExp2*, and *Yr26*; homozygous for its virulence phenotypes to *Yr1*, *Yr2*, *Yr3*, *Yr6*, *Yr7*, *Yr8*, *Yr9*, *Yr17*, *Yr25*, *Yr27*, *Yr28*, *YrA*, *Yr32*, and *Yr47*; and heterozygous for its VPs to *Yr1*, *Yr2*, *Yr9*, *Yr17*, *Yr47*, *YrA*, *YrSp*, *YrTr1*, *YrExp2*, and *Yr47*. Heterozygosity and homozygosity of avirulence/virulence phenotypes have been reported using selfed populations of *PST* isolates of Chinese races [27,28], a US race of *PST* [41], and a Chinese predominant race C*YR32* of *PST* [7]. We found that both *Yr5* and *Yr15* genes were resistant against all the progeny isolates and the parental isolate, indicating their effectiveness in China and Pakistan, which correlates with previous studies [7,27,28,41]. In addition to the avirulence phenotypes produced by these two genes, avirulence genes to eight other *Yr* single-genes (*Yr10*, *Yr24*, *Yr32*, *YrSp*, *YrTr1*, *YrTye*, *YrExp2*, and *Yr26*) were also homozygous in this study. Similarly, Tian et al. [27,28] have reported *Yr24*, *Yr26*, and *YrTr1* as avirulent to the Chinese race of *PST* and its progeny population. Through virulence testing of the progeny isolates, Yuan et al. [41] found homozygous avirulence phenotypes for *Yr10*, *Yr24*, *Yr32*, and *YrTr1*. The homozygous avirulence phenotypes to *Yr10*, *Yr24*, *Yr26*, *Yr32*, and *YrTr1* have also been reported [7].

The segregation of avirulence/virulence phenotypes on 10 *Yr* single-gene lines determined that the VPs to *YrSp*, *YrTr1*, and *Yr47* were controlled by a single locus; those on six *Yr* single-gene lines (*Yr1*, *Yr2*, *Yr9*, *Yr17*, *Yr27*, and *YrA* were controlled by two loci. Both single-locus and two loci have been reported in previous studies [7,27,28,41]. Tian et al. [27] reported one locus controlling virulence phenotypes to *YrSp*, which is similar in this study. In another study, Tian et al. [28] reported avirulence/virulence phenotypes to *Yr1*, *Yr7*, *Yr8*, *Yr27*, and *YrExp2* controlled by one locus and avirulence/virulence phenotypes to *Yr2*, *Yr*4, *Yr6*, *Yr9*, *Yr28*, *Yr32*, *Yr44*, *Yr7*6, and *YrTr1*, which is similar in case of *Yr1* controlled by a single locus, and *Yr2* and *Yr9* controlled by two loci; however, on the other hand, it contradicts with *YrTr1*, which is controlled by single locus in this study. A single locus controlling the VPs to *Yr1*, *Yr6*, *Yr7*, *Yr8*, *Yr9*, *Yr25*, *Yr27*, *Yr28*, and *YrA* (*Yr7*3 and *Yr7*4) and two loci controlling the VPs to *Yr2*, *Yr17*, *Yr32*, *Yr44*, *Yr7*6, and *YrExp2* were reported [41]. However, Wang et al. [7] found that the VP to *YrExp2* was controlled by a single gene, which is contradicting with results of Yuan et al. [41] as well as the results of this study. The gene-for-gene hypothesis [42] supports the concept of one-locus controlling *Yr* single-gene lines, and by contrast, two loci controlling avirulence/virulence phenotypes in some of the other *Yr* single-gene lines [7,27,28,42]. Two loci may regulate a single gene, which can be explained using additional genes in the wheat *Yr*-gene lines as previously discussed [7]. Previous studies and in this study, the resistance genes to *YrA* were determined to be controlled by two complementary genes [7,43]. Based on this study and previous studies, it is concluded that the interaction between resistance genes in wheat *Yr* gene-lines and corresponding genes in the *PST* pathogen controlling avirulence follow gene-for-gene hypothesis.

In this study, we determined that avirulence/virulence phenotypes were controlled by dominant or recessive genes as previously described. A single dominant gene was controlling avirulence to *Yr6*, *Yr32*, and *YrSp* [27], and to *Yr7*, *Yr8*, *YrSp*, and *YrExp2* [28]. However, more dominant genes controlling virulence were determined [7,27,28,41]. In this study, we determined genes controlling avirulence to *YrSp*, *YrTr1*, and *Yr47* controlled by a single dominant gene, and two recessive genes were controlling phenotypes to *Yr1*, *Yr2*, *Yr9*, *Yr17*, *Yr27*, and *YrA*, which correlates with the previous studies with all dominant genes for avirulence [41]. According to Wang et al. [7], dominant virulence genes provide advantages to the obligate parasite for survival and growth on the host plants, but without variation in the *PST* races, it is not possible to overcome race-specific resistance genes present in the host cultivars. It emphasizes the cloning of dominant genes for virulence and their molecular interaction with resistance genes. The effectiveness of *Yr5* and *Yr15* resistance genes both in Pakistan and in China is observed in this study and previous similar studies. The genetic studies and inheritance of progeny populations of Chinese-, American-, and Pakistan-dominant races of *PST* are useful to understand the virulence diversity in the pathogen population and their ultimate use in the breeding programs for resistant wheat cultivars. 

A comparison of inheritance of avirulence/virulence of the population of Pakistani dominant race of *PST* with previous studies show that the avirulence to *Yr1* was found to be dominant in the Chinese population [27] and a US population of *PST*v-11 [41], which is inconsistent with this study. Observations of change in recessive to dominant avirulence for *YrExp2*, our results are also inconsistent with Tian et al. [28] and Wang et al. [7]. Similarly, for *Yr9*, two complementary recessive genes (1:15 ratio) for avirulence were detected [7,41], as well as showing switching of dominance to recessiveness. This shows the involvement of different alleles controlling phenotypes and the alleles at one locus behave differently with the alleles at another locus; this indicates that different *PST* isolates have different avirulence/virulence phenotypes due to their different alleles controlling phenotypes. Studies on the genetics of *PST* have identified single and multiple alleles controlling avirulence/virulence phenotypes in different wheat *Yr* gene lines and the complex interaction between alleles at different loci need to be studied by cloning the alleles controlling avirulence/virulence and their interaction with corresponding resistance genes [7]. 

The use of resistance genes in breeding programs needs to determine which genes are resistant to dominant races of the pathogen, and it is only possible through studies on the inheritance of avirulence/virulence genes. In this study, a total of 60 multilocus genotypes were identified after clonal correction. The heterozygosity of 10 SSR loci ranged from 45% to 65% with an average of 56%. The 10 SSR markers fit a 1:2:1 ratio for homozygous (a1a1): heterozygous (a1a2): homozygous (a2a2) genotypes (Supplementary Scheme S1) for each locus (*p* = 0.06 to 0.30). The data indicated that only 10 markers were suitable to construct a linkage map. Linkage 1 consisted of nine SSR markers, and linkage 2 consisted of one SSR marker and 10 avirulence/virulence genes. Dominant virulence (or recessive avirulence genes) *avir10* and *avirSp* were closely linked with each other (3.90 cM), which indicates their possible presence on the same chromosome. Although, at the other end, *Avir17* and *M2* (*Pst*P008) were closely linked with each other at almost the same distance (3.90 cM). The avirulence/virulence genes *VYr9*, *Avir17* and *Avir47* were closely linked to the marker M2 (*Pst*P008), indicating the clustering of these genes at a specific location on the chromosome. 

Homozygosity for avirulence phenotypes of both *Yr5* and *Yr15* was observed in this study, and the same results have been reported in similar studies [7,27,28,41]. These results indicate that these two genes (*Yr5* and *Yr15*) are still effective against *PST* not only in Pakistan and China, but in almost all wheat-growing areas in the world [44]. Due to their effectiveness against *PST* races, both *Yr5* and *Yr15* are selected as differentials in the breeding programs [45]. If the resistance gene has a corresponding avirulence gene in the homozygous parent, the durability of the resistance gene becomes longer as compared to a corresponding resistance gene with a heterozygous avirulence gene. According to Yuan et al. [41], the mutations cycle is required two times to convert avirulence to virulence if avirulence is dominant, and they identified the homozygous avirulence genes to *Yr8*, *Yr10*, *Yr24*, *Yr26*, *Yr32*, and *YrTr1*. Tian et al. [27] reported the homozygous avirulence to *Yr8* in the progeny population generated by selfing a Chinese isolate, Pinglan 17–7. In this study, high genetic diversity among the progeny isolates obtained from artificially inoculated Himalayan *Berberis* spp., is observed under controlled greenhouse conditions.

It is found that through sexual reproduction, a large number of genotypes and phenotype patterns can be generated [7,27,28,41]. From one-hundred-and-fifteen progeny isolates, a total of 25 VPs were identified, some of which had a broader virulence spectrum. These results showed that a sexually produced progeny population had a wide range of virulence phenotypes with more virulence as compared to the parental isolate. More than 56% of progeny isolates had a broader virulence spectrum as compared to the parental isolate. Previous studies, and the results of the present study, support the hypothesis of the role of sexual reproduction in pathogenic diversity as compared to asexual or clonal reproduction [28,46]. All these studies are in agreement with the assumption that sexual reproduction, if it occurs in natural conditions, causes the production of a large number of virulent races of *PST*. However, as previously mentioned by other studies [7,27,28], it is still not clear that sexual reproduction plays a vital role in pathogenic diversity under natural conditions. For example, *P*. *striiformis* f. sp. *tritici* aecia was not identified on barberry plants under natural conditions in the United States, and only *P*. *graminis* f. sp. *tritici* or other formae speciales were found on grasses [47,48]. Barberry phenology and degradation of teliospores were the main causes of lack of sexual reproduction of *P*. *striiformis* f. sp. *tritici*. However, favorable weather conditions in northwestern China make it possible for teliospores of this fungus to survive in the winter and cause infection on barberry plants in the spring, as later on, aecia of *P*. *striiformis* f. sp. *tritici* were recovered from the naturally infected barberry species in this area [49]. However, under natural conditions, a shallow frequency of stripe rust fungus was found by Wang et al. [21] and Zhao et al. [18], suggesting that sexual reproduction takes place in China under natural conditions in some areas. They found a relationship between the two isolates obtained from barberry plants and a predominant race C*YR32* present in the surrounding wheat fields in that area. However, in different parts of the world, the role of alternate hosts and sexual reproduction in generating a diversity of the stripe rust fungus still needs to be explored; especially in the areas of high pathogenic diversity like in *PST* over-summering areas in China (Gansu region) and in the Himalayan region in Pakistan and its surrounding regions near China and Nepal [50,51,52].

After the discovery of alternate hosts of wheat stripe rust, the role of sexual reproduction has become more valuable, and the approach of artificial inoculation of these alternate hosts to study the genetics of this fungus has attained the attention throughout wheat-growing areas in the world [52,53]. A study about the role of sexual reproduction, using selfing populations of *P*. *striiformis* f. sp. *tritici*, was done by Wang et al. [25]. They used the most virulent isolate of *PST*-127 and identified homozygous virulences to *Yr1*, *Yr2*, and *Yr9* and homozygous avirulence to *Yr5*, *Yr15*, *Yr24*, *Yr32*, and *YrSp*. They found that a dominant gene was controlling virulences to *Yr6*, *Yr7*, *Yr8*, *Yr19*, *Yr27*, *YrExp2*, and *YrTye*, whereas *Yr44* was controlling virulences by two dominant genes; one recessive gene was controlling *Yr10*, *Yr17*, *YrTr1*, and *YrExp1*; and an unknown gene by two recessive genes was controlling *Yr32*. In this study, we also found that avirulence to *YrExp2* was controlled by a dominant gene. In contrast, we found that avirulence to *Yr6* was controlled by a dominant recessive gene. The virulences to *Yr1* and *Yr9* of the progeny isolates were controlled by two complementary recessive genes. For virulence to *Yr2*, *Yr17*, *Yr47*, and *YrA*, the avirulence was controlled by a recessive gene and *Yr44* by two recessive genes. These results suggested that the avirulence/virulence to a *Yr*–single gene line can be dominant or recessive, and different genes can control it. These results correlate with the results of previous studies [7,27,28,41]. Generally, the results of this study are in agreement with the gene for gene hypothesis [44], with some exceptions. For other fungal species, the concept of one-for-more genes ere previously reported by Statler [54,55,56,57]. There are two reasons, suggested by Tian et al. [27], for exceptions for the typical gene-for-gene concept: the presence of an additional modifier gene and presence of more than one resistance genes in the wheat lines. It is easy to determine the inheritance modes of avirulence/virulence in general; however, more studies are needed to discover the particular avirulence/virulence [58].

Resistance gene *Yr1* was the first identified gene against *PST* but now virulence to *Yr1* with different frequencies has been reported in various countries [59]. Similarly, the virulences to *Yr9* and *Yr17* are generally high throughout the world [39]. The significant causes of stripe rust epidemics in many countries of the world were virulences to *Yr17* and *Yr27*, during the last two decades [8]. Although we found *YrSp*, *YrTr1*, *YrTye*, and *Yr10* as resistant genes against all progeny isolates and the parental isolate, the virulence frequencies to *Yr10* are generally low, or it varies from country to country [39]. The single-gene lines with resistant genes, *YrSp*, *YrTr1*, and *YrTye*, have been used in differential sets, especially in the United States, due to their high differential ability [60]. The virulence to *YrSp* was very rare in the United States, before 2000, but later becomes significant, and recent studies showed virulences to *YrSp* found in isolates from China, Turkey, and Uzbekistan, but not in many other countries in the world. The virulence frequencies to *YrTr1* and *YrExp2* vary significantly from state to region. 

## 4. Materials and Methods

### 4.1. PST Isolate, Isolation of Single Urediniospore, and Teliospore Production

**Figure 3:**
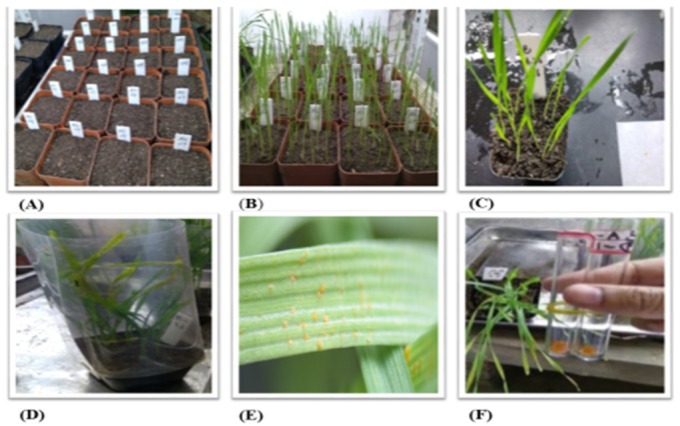
(**A**) Isolation and multiplication of urediniospores of *PST* and (**B**) growing of wheat seedlings of susceptible wheat variety “Mingxian 169”. (**C**,**D**) Inoculation of 10-day-old wheat seedlings covered with plastic cylinder, (**E**) appearance of urediniospores 10 days after inoculation (dai), and (**F**) collection of urediniospores in glass tubes.

The wheat leaves bearing urediniospores of a dominant *PST* race 574232 were collected from the experimental plots of the Barani Agricultural Research Institute, Talagang Road near Balkassar Interchange M–2 (32°55′49″ N 72°51′20″ E, El. 498m) district Chakwal during March to April 2016 [19,30]. These samples were kept in paper bags and brought in to the lab at Northwest A&F University, China, and kept at 4 °C for further use. The single urediniospores (SU) were isolated following the methods described by Mehmood et al. [19]. The SU isolate [Pak–1–(A)–9] was used to inoculate four adult wheat plants of Mingxian 169 for the production of teliospores. Adult plants were inoculated at the growth stage 50 [61] and kept at 10 °C for 24 h in a dew chamber at 90–99% RH and then transferred to another growth compartment at 16 °C with 16/8 h light–dark cycle. To accelerate the formation of teliospores, the plants were moved to another growth chamber at 25/16 °C day/night when there was maximum sporulation [62]. Wheat leaves bearing telia were harvested approximately 35 to 40 days after inoculation (dai), and kept at room temperature for two days and then stored at 4 °C for further use. The procedure is shown in Figure 3.

### 4.2. Growing Barberry Plants, Artificial Inoculation, and Selfing

The young plants of susceptible Himalayan barberry (*B. pseudumbellata*) were grown under controlled greenhouse conditions and inoculated with germinated teliospores of a Pakistani dominant race (574232) of *PST* using the method as described by Mehmood et al. [19]. Two-day-old wheat leaf segments bearing *PST* telia, soaked in distilled water in a petri dish (25 cm in diameter), were placed on 2% water agar media and incubated at 10 °C in the dark. After successful germination of teliospores (12 to 24 h after planting), the water agar plates having basidiospores were used to inoculate fresh leaves of barberry plants. A total of 150 barberry plants were inoculated. The plates were inverted on barberry plants covered with a plastic cylinder. The inoculated plants were incubated at 100% RH for 3 to 4 days at 10 °C in the dark. The inoculated plants were shifted to a spore-proof growth chamber with 90 to 100% RH, a diurnal cycle of 16/13 °C, and 12/12 h light–dark to promote pycnial formation. Plants were observed and misted with water every day until pycnia appeared (12 to 14 dai) on the upper surface of the leaves. Selfing was done by picking pycnial nectar from one pycnium and delivering to another using a sterilized toothpick. Approximately 18 to 22 dai, when aecia appeared on the abaxial surface of leaves, the RH was lowered to 60–70% to stop the opening of aecial cups [26]. 

### 4.3. Progeny Population

From artificially inoculated Himalayan *Berberis* spp., aecial cups (2–3 mm in length) were selected to inoculate 10-day-old wheat seedlings of Mingxian 169. The single aecial cup from a leaf was gently cut with sterilized blade placed on the glass slide having 1 to 2 drops of distilled water and crushed with a sterilized fine needle to release aeciospores. The suspension containing aeciospores from a single aecial cup was used to inoculate one leaf seedling of susceptible wheat after removing wax on leaves and misting with distilled water. Each wheat seedling was separated with plastic cylinders with an open top to prevent it from contamination. The inoculated wheat seedlings were kept at 10 °C for 24 to 36 h in a dew chamber for incubation at 100% RH. The seedlings were transferred to a spore proof growth chamber having 16 °C temperature and 16/8 h light–dark periods. To get a pure single uredinial isolate (SU), the single lesion was used from the wheat leaf before the breakage of the wheat epidermal layer, to inoculate Mingxian 169 seedlings using the procedure with some modifications [27,28]. A total of one-hundred-and-fifteen single urediniospores (SU) isolates were recovered and multiplied to get enough quantity for avirulence/virulence tests.

### 4.4. Virulence Phenotyping on Wheat Yr Single-Gene Lines

Virulence phenotypic tests were conducted using one-hundred-and-fifteen progeny isolates (SA), on a set of 24 wheat *Yr* single-gene lines (which were Avocet Susceptible, nearly isogenic lines) [27,28] (Supplementary Table S1) to test the virulence variation. Ten centimeter diameter plastic pots were used to grow the sets of wheat gene lines, having commercially mixed peat moss and soil. In each pot, 7–8 seeds of each wheat *Yr* single gene line were planted at four corners of the pot and kept in a rust-free greenhouse chamber. At approximately day 12, seedlings at the 2-leaf stage were used to inoculate with the fresh suspension of each isolated single uredinia (SU) and parental isolate with a small sprayer machine. Mingxian 169 variety was used as a susceptible check. The inoculated wheat seedlings were kept at 10 °C for 24 h in a dew chamber, and after that, the seedlings were transferred into a growth chamber having 16 °C temperature with 16/8 h light–dark period as described above. Infection types (ITs) data were recorded 18–20 dai, using a 0–9 scale [63]. Plants having ITs 0–6 were considered avirulent (A) and plants having ITs 7–9 virulent (V). 

### 4.5. DNA Extraction

DNA was extracted directly from dried urediniospores of one-hundred-and-fifteen progeny isolates using the method, with some modifications, as described by Aljanabi and Martinez [64]. The fresh urediniospores (40 mg of each progeny isolate) were transferred into a centrifuge tube (2 mL), having two steel balls (4 mm in diameter), and grinded at 30 rpm for 6 min in a swing mill to become fine powder (Tissue Lyser II, QIAGEN, Hilden, Germany). After grinding, 800 μL preheated cetyltrimethylammonium bromide (CTAB) extraction buffer (0.4 M NaCl; 10 mMTris HCl, pH 173 8; 2 mM EDTA pH 8) was added, blended the solution in the tube gently, and incubated at 65 °C in water bath for 1 h, and each tube was shaken gently after every 10 min. After water bath, 500 μL phenol–chloroform–isoamyl alcohol (25:24:1 by volume, pH 7.0–8.0) was added and centrifuged at 12,000 rpm for 10 min at 4 °C. The supernatant aqueous solution (400–450 μL) was transferred into a new centrifuge tube (2 mL) and 500 μL chloroform was added and centrifuged at 12,000 rpm for 10 min. The supernatant (300 μL) was transferred to a fresh centrifuge tube (1.5 mL), and 500 μL pre-cooled isopropanol was added and kept at −20 °C for 24 h. The solutions were centrifuged at 12,000 rpm at 4 °C for 30 min. After removing the supernatant, the DNA pellet was washed with 75% ethanol (800 μL) and centrifuged at 12,000 rpm for 10 min. The DNA pellet was washed twice with 75% ethanol and then air-dried for 30 min at room temperature. The DNA pellet was dissolved in 50 μL 1 X TE buffer solution (10 mM Tris–HCl and 1 mM EDTA, pH 8.0). One microliter of RNase was added and kept at 37 °C for 1 h to digest the RNA completely. The RNase was removed, and purified DNA was dissolved in TE buffer by precipitating, centrifuging, and resolving as described above. DNA was quantified using an ND-1000 spectrophotometer (Bio-Rad, CA, USA), and the DNA solution was diluted to 50 ng/μL for further use in polymerase chain reaction (PCR) tests with simple sequence repeat markers, and the rest of extracted DNA was stored at –20 °C.

### 4.6. Simple Sequence Repeat (SSR) Markers

A set of one-hundred-and-forty-one pairs of SSR primers, specific for *PST* (obtained from Shanghai Sangon Bio-Tech Company, Shanghai, China), were screened for SSR genotype characterization of parental Pakistani isolate and one-hundred-and-fifteen progeny isolates of *PST* (Supplementary Table S2).

### 4.7. Polymerase Chain Reaction (PCR) Amplification and SSR Genotyping

The PCR assays were performed using a thermal cycler {(PTC400, Bio-Rad) S1000, Bio-Rad)} with the conditions as described by Wang et al. [7]. Each PCR reaction was performed in a total of 25 μL volume (Taq Mix 12.5 μL, ddH_2_O 8.5 μL, Primer F 1 μL, Primer R 1 μL, sample DNA 2 μL). The touchdown PCR amplification conditions were used as follows; 95 °C for 5 min, 10 cycles of 94 °C for 45 s, 64 °C for 45 s (with reduction of 1 °C at each cycle), and 72 °C for 45 s; followed by 25 cycles with 94 °C for 45 s, 54 °C for 45 s, and 72 °C for 45 s; and one final extension at 72 °C for 10 min. To ensure that there was no contamination, in control reactions, template DNA was substituted by sterile distilled water. The PCR products were analyzed using a 3730XL DNA Analyzer (Applied Biosystems, Waltham, MA, US). SSR amplicons were scored using software Gene Marker HID [65].

#### Urea–PAGE Gel, Electrophoresis, and Silver-Staining

The urea–PAGE gel (6%) solution was prepared using ultrapure urea 40% (420 g), polyacrylamide solution (19:1) 150 mL, 10 X TBE solution (Tris–Borate, EDTA buffer) 100 mL, to make the volume 1 L. The method was followed with some modifications as described by Chen et al. [66]. After the gel solidified, the apparatus was set up, and 1 X TBE buffer was filled into the lower chamber that the glass plates were submerged 2–3 cm with buffer. Similarly, the upper chamber was also filled with buffer up to the top of the gel. The air bubbles were carefully removed from the wells using a pipette.

Each PCR amplification product and a molecular size marker (M) (DL 2000, TaKaRa Bio Inc. Japan) was injected (6 µL each) into the wells of polyacrylamide gel (acrylamide-bis-acrylamide 29/1) in buffer 1X TBE (2 mM EDTA, 10 mM Tris–borate, pH 8.0). The upper lid was attached carefully, and the cables were plugged to a high voltage battery and electrophoresed at 1400 V for 2.0 to 2.5 h. When the dye font reached the end of the gel slides, the voltage was stopped, and the slides were removed out carefully from the apparatus. The upper glass slide was detached from the gel slide and washed with ddH_2_O in a large steel dish for a few seconds. Then, the slide was placed in a solution (2 L ddH_2_O, 2g Ag NO_3_) for 10 min, shaking every 2 min. After that, the gel was washed again in ddH_2_O for 8 s. The gel was washed into the second solution (32 g NaOH, 3 mL formaldehyde, and 2 L ddH_2_O) for 8 to 10 min. The gel was immediately removed from the solution when the desired bands appeared. The gel was kept in 0.75% Na_2_CO_3_ for 1 min and washed again in ddH_2_O for few seconds. The gel was dried at room temperature for ~1 h. A set of 10 SSR markers were selected from a total of one-hundred-and-forty-one SSR primers used for screening purposes. The list of selected SSR markers and range of sizes of their band lengths in base pairs (bp) are given in Supplementary Table S3.

### 4.8. Data Analysis

Virulence phenotypes of the progeny isolates were determined using software VAT version 1.0 (https://en-lifesci.tau.ac.il/node/3094/done?sid=1581&token=3d97a66eda1a93bc3e8af4deec679924) [67]. Chi squared test (χ^2^) was used to assess the goodness-of-fit for the observed segregations of avirulence versus virulence phenotypes and SSR alleles to the expected ratio of a single locus [6]. The correlation coefficient between VPs and SSR genotypes was calculated using NTSYS–pc (version 2.10) MX COMP (https://ntsyspc.software.informer.com/2.1/). The linkage maps were built using QTL IciMapping software (version 4.1) (http://www.isbreeding.net/software/?type=detail&id=18) [68]. The threshold of the logarithm of odd (LOD) score was set as 3.00 after a permutation test. 

## 5. Conclusions

In this study, the genetic inheritance of a selfing population of 115 isolates revealed that a single isolate of a dominant race of *PST* could produce a large number of races with high virulence diversity. The results show high genetic diversity in the progeny isolates due to sexual reproduction on the Himalayan barberry under controlled greenhouse conditions, which suggests a vital role of alternate hosts in generating virulence diversity under natural conditions in the Himalayan region in Pakistan near the border of China and Nepal.

## Figures and Tables

**Table 1:** Virulence phenotypes (VPs) of the parental and one hundred and fifteen progeny isolates of dominant Pakistani race (574232) of *Puccinia striiformis* f. sp. *tritici* (*PST*) on 24 wheat *Yr* single-gene lines.

VPs **	No. of isolates	Avirulence (A) and Virulence (V) on Wheat *Yr* Single-Gene Lines *									
		*Yr*1	*Yr*2	*Yr*9	*Yr*17	*Yr*27	*Yr*A	*Yr*Sp	*Yr*Tr1	*YrExp*2	*Yr*47
Parental isolate	N/A ***	V	V	V	V	V	V	A	A	A	A
1	42	V	V	V	V	V	V	A	A	A	A
2	25	V	A	V	V	V	V	A	V	A	A
3	5	V	V	A	V	V	V	A	A	V	A
4	3	V	V	V	A	A	V	A	A	A	V
5	1	V	V	V	V	V	V	V	A	A	A
6	1	V	V	V	V	V	A	A	A	A	A
7	4	A	V	A	A	V	V	A	A	A	A
8	2	V	V	V	V	V	V	V	A	A	V
9	1	A	V	V	V	V	V	A	V	V	V
10	3	V	V	V	V	A	V	V	A	V	A
11	1	V	V	A	V	V	V	A	A	A	V
12	2	V	V	V	V	V	V	V	V	A	A
13	1	V	V	V	V	V	V	A	A	A	V
14	1	V	V	V	V	V	A	A	A	V	A
15	2	V	V	V	V	V	V	V	V	A	V
16	1	V	A	V	V	A	V	V	A	A	A
17	7	V	V	V	V	V	A	V	V	V	V
18	3	A	V	V	V	V	V	V	A	V	A
19	3	A	A	V	V	V	V	A	A	V	V
20	2	V	V	V	V	V	V	V	A	A	A
21	1	V	V	V	V	V	V	A	A	A	V
22	1	V	V	A	V	V	V	A	A	A	A
23	1	A	V	V	V	V	V	A	A	V	A
24	1	V	V	V	V	V	V	A	A	A	A
25	1	V	V	V	V	V	V	A	A	A	A

* The parental Pakistani isolate of a dominant race (574232) of *Puccinia striiformis* f. sp. *tritici* (*PST*) and 115 progeny isolates were tested on 24 *Yr* single-gene lines. The parental and progeny isolates were all avirulent (ITs 0–6) to *Yr5*, *Yr10*, *Yr15*, *Yr24*, *Yr32*, *Yr43*, *YrSp*, *YrTr1*, *YrExp2*, *Yr26*, and *YrTye*; and virulent (ITs 7–9) to *Yr1*, *Yr2*, *Yr6*, *Yr7*, *Yr8*, *Yr9*, *Yr17*, *Yr25*, *Yr27*, *Yr28*, *YrA*, *Yr44*, and *Yr3*. ** Virulence phenotypes. *** Not applicable.

**Table 2:** Infection types, avirulence/virulence segregation in the progeny isolates obtained from selfing a Pakistani isolate of *Puccinia striiformis* f. sp. *tritici* on wheat *Yr* single-gene lines, and their probability (*P*) values of Chi squared tests for goodness of fit to the observed to the theoretical ratios.

Wheat *Yr* Single-Gene Lines	Infection Type of Parental Isolate	Observed No. of Progeny Isolates		Expected Ratio (V/A)	χ^2^	^a^ *P*	*Virulence Genes*
		Avirulent	Virulent				
*Yr*1	8	8	107	1:15	2.63	0.06	*VYr1–1, VYr1–2*
*Yr*2	9	9	106	1:15	1.58	0.14	*VYr2–1, VYr2–2*
*Yr*9	9	11	104	1:15	1.55	0.14	*VYr9–1, VYr9–2*
*Yr*17	8	7	108	1:15	1.60	0.13	*VYr17–1, VYr17–2*
*Yr*27	9	7	108	1:15	1.60	0.13	*VYr47–1, VYr47–2*
*Yr*A	9	9	106	1:15	1.58	0.14	*VYrA–1, VYrA–2*
*Yr*Sp	1	30	85	1:3	0.76	0.09	*VYrSp*
*Yr*Tr1	1	28	87	1:3	0.62	0.27	*VYr Tr1*
*Yr* Exp2	1	64	51	7:9	0.15	1.96	*VYrExpt2–1, VYrExpt2–2*
*Yr*47	1	26	89	1:3	0.65	0.07	*VYr10*

χ^2^, chi squared test for goodness of fit between expected and observed numbers of progeny isolates in the avirulent and virulent groups to the theoretical ratios. ^a^ *P*, probability greater value due to chance alone. *p* ≥ 0.05 indicates that the two genes are independent and not linked; *p* < 0.05 indicates that the two genes are linked.

## Supplementary Materials

Supplementary materials can be found at https://www.mdpi.com/1422-0067/21/5/1685/s1.
