# Supplementary material for: Study of Inheritance and Linkage of Virulence Genes in a Selfing Population of a Pakistani Dominant Race of Puccinia striiformis f. sp. tritici
Source: Int J Mol Sci. 2020 Feb 29;21(5):1685. doi: 10.3390/ijms21051685 (PMC7084513; doi:10.3390/ijms21051685)
Supplement: Supplementary file 1 [file ijms-21-01685-s001.pdf]

Supplementary Material

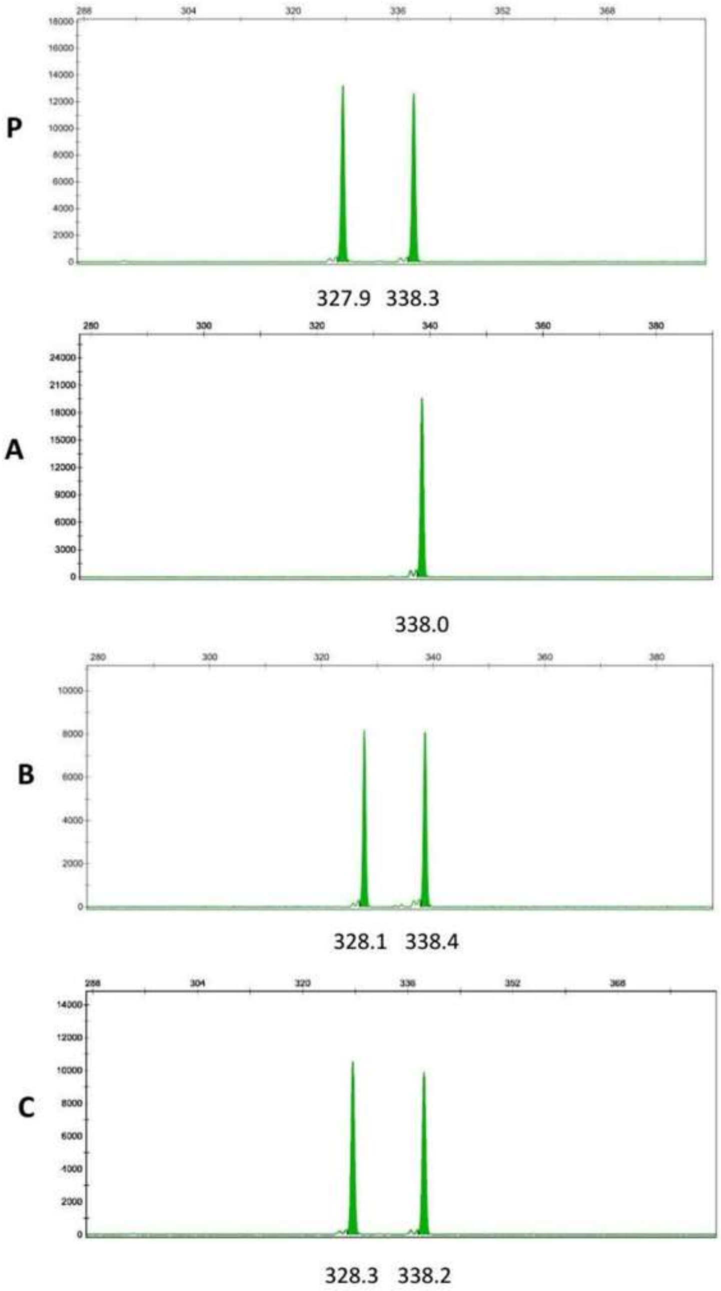

Supplementary Figure 1. The heterozygous and homozygous amplicons produced by SSR markers SUNI*Pst*11–10 in the parental isolate and three progeny isolates; **P**: parent isolate, **A**: isolate 23, **B** isolate 98, and **C**: isolate 59.

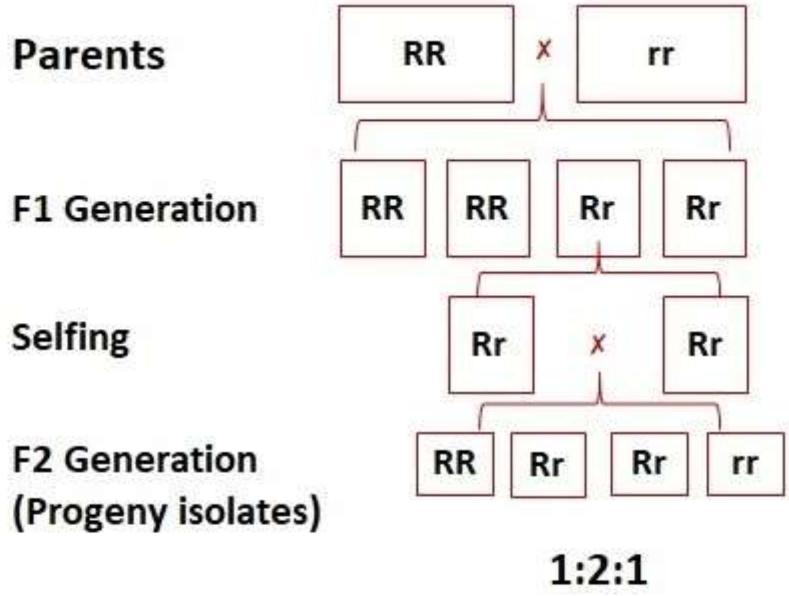

Supplementary Scheme I. Mendel's law of inheritance.

Supplementary Table S1. Wheat *Yr* single–gene lines used to characterize the avirulence/virulence phenotypes (VPs) of Pakistani isolate and its progeny isolates of *Puccinia striiformis* f. sp. *tritici*

**Line**	***Yr* gene**	**ID number**	**Habit**
AvS*Yr*1NIL	*Yr1*	WG00003	Spring
AvS*Yr*5NIL	*Yr5*	WG00004	Spring
AvS*Yr*6NIL	*Yr6*	WG00014	Spring
AvS*Yr*7NIL	*Yr7*	WG00005	Spring
AvS*Yr*8NIL	*Yr8*	WG00006	Spring
AvS*Yr*9NIL	*Yr9*	WG00007	Spring
AvS*Yr*10NIL	*Yr10*	WG00008	Spring
AvS*Yr*15NIL	*Yr15*	WG00009	Spring
AvS*Yr*17NIL	*Yr17*	WG00010	Spring
AvS*Yr*24NIL	*Yr24*	WG00016	Spring
AvS*Yr*27NIL	*Yr27*	WG00018	Spring
AvS*Yr*28NIL	*Yr28*	WG00032	Spring
AvS*Yr*32NIL	*Yr32*	WG00031	Spring
AvS*Yr*43NIL	*Yr43*	WG00097	Spring
AvS*Yr*SPNIL	*YrSP*	WG00019	Spring
AvS*Yr*Tr1NIL	*YrTr1*	WG00020	Spring
Avs/Exp1/1–1Line 74	*YrExp2*	WG00099	Spring
AvS*Yr*ANIL	*YrA*	WG00002	Spring
Kalyasona	*Yr2*	PI410916	Spring
Hugenoot	*Yr25*	PI 591944	Spring
92R137	*Yr26*	–	Spring
Tyee	*YrTye*	CI017773	Winter
Vilmorin 23	*Yr44*	PI 125093	Winter
Hybrid 46	*Yr4*	PI 164755	Winter

Supplementary Table S2. List of simple sequence repeat (SSR) markers withsequences.

No.	Primer	(5'to3')
1	PstP006-F	GTTTGATTTTCCCTATGC
	PstP006-R	AACTGAACGGAAGATGC
2	PstP007-F	GATTTGCGAGGTCACTTT
	PstP007-R	TGGTTGTGATAACGATGA
3	PstP008-F	CCCTTGAGTAGTATGACC
	PstP008-R	AGAAGAGGACGAGAAGAT
4	PstP021-F	CCTCGACGCCCTCATTC
	PstP021-R	TTGGTGACGAGCAGGTAT
5	PstP025-F	ATGTAAATGTAGCACCAAAC
	PstP025-R	TCATGCTCGGTATGTCTC
6	PstP027-F	CAGCGTAACTCCCAGGAT
	PstP027-R	GACCGTGTTCAGCCAAGT
7	PstP028-F	GCATTCAAACAGCAGCAA
	PstP028-R	GGTTAGGGTATGGCAAGG
8	PstP029-F	ACAATCCTCAAGGTGGTG
	PstP029-R	GTTCGCTTTGTTGGTTAT
9	PstP030-F	AAGGAAAAGAACTGTATG
	PstP030-R	TTCAGATGCTCTATTCAA
10	PstP031-F	TTGGGCGTCCTGGCATTG
	PstP031-R	ACCCGTTCCTTCTTGGTCTTGC
11	PstP033-F	ACAGAAGGAAGGCAGATT
	PstP033-R	GGGGTTTGATGTTATTAC
12	PstP034-F	CCTCTTTTGTCCGCTTCC
	PstP034-R	GTGCGACATGGTTTGACATT
13	SUNIPst05-47-F	TGTTGCAGTAGATTGTGAAGATG
	SUNIPst05-47-R	CTGGGATTGAGCTGGATATG
14	SUNIPst09-06-F	GGGCCCAATCACCTGTCTATAA
	SUNIPst09-06-R	CAGGCTTGAGGTGGTTTGAAG
15	SUNIPst10-48-F	CCGGGTTTGCACTCTTTGTC
	SUNIPst10-48-R	TGATTGCCCTGACTGTGAGC
16	SUNIPst09-48-F	AGCACCCCAACAATCATCACA
	SUNIPst09-48-R	CCGAGGGTGAGTTTGGTTGA
17	SUNIPst10-06-F	GCGGCATTAGCGTCTCTTC
	SUNIPst10-06-R	TCGCTTTCGTTCTCCATTGTC
18	SUNIPst13-42 -F	CTGCCCTTGTATTCCTCTTGTG
	SUNIPst13-42 -R	CACCACCACCAATAATGATAAA
19	SUNIPst05-39-F	GCTCGAAACTGGGTCTGATG
	SUNIPst05-39-R	ACGGCGACCTTCCAACATCT

20	SUNIPst11-10 -F	CTGGTTTTGCTTTTAGGAGTTT
	SUNIPst11-10 -R	AGGCAATGGTCCAGAAAAGAGT
21	SUNIPst11-44 -F	GAACCGATCAGAACATTGAGAATA
	SUNIPst11-44 -R	CTCTATCTCAATCACCCTTTCTATT
22	SUNIPst09-40 -F	AGGGAGTTGATAAGGTTGTTGA
	SUNIPst09-40 -R	CTCCTGCTCAAACCAAAGAGA
23	SUNIPst15-26 -F	TGNACCACTTCAGGCGAAAAGA
	SUNIPst15-26 -R	AAGGGCCTCGTCTAAGAATAAA
25	SUNIPst16-42 -F	CCCGCCAGGCTAGAAATAGAA
	SUNIPst16-42 -R	AGCCGATCCTCCAAAACTATCC
26	SUNIPst09-19 -F	GTCGAGGTGATGGCGGTAAA
	SUNIPst09-19 -R	GCGTCGAAGTTCAAGAAGGTTT
27	SUNIPst11-04-F	AAGGGTCTGAAGAAGAAACACA
	SUNIPst11-04-R	ACCACCCATGAGTCTCTATTCC
28	SUNIPst11-21-F	TGGTTTGCTGTGAAAAGTATTG
	SUNIPst11-21-R	TGTCGCATACTTGGATGAATAG
29	SUNIPst15-30 -F	GACGTATCGATGAGTGTGGAGA
	SUNIPst15-30 -R	TAGCCAGTGCTCAAGATAACGA
30	SUNIPst17-23 -F	TGCAGGCTGAGTAGAACACCAA
	SUNIPst17-23 -R	CGCCCACCTTCACATAGAATG
31	SUNIPst17-34 -F	CACTACCAAACATGTACACTGTACC
	SUNIPst17-34 -R	GCTCGATCAGTACCTCNGTTTC
32	SUNIPst09-17 -F	TTAGGGTTCACAAGTGCCGTT
	SUNIPst09-17 -R	CCGCTATCAACCTCCAAAATCA
33	SUNIPst13-15 -F	TGNAGGACCGGTTCGAGATAAA
	SUNIPst13-15 -R	AGGGACTGGAATNCTAGGTTTTT
34	CPS08 -F	GATAAGAAACAAGGGACAGC
	CPS08 -R	CAGTGAACCCAATTACTCAG
35	CPS09-F	CGGGAGAAGACCTGAGC
	CPS09-R	AGAAAACGGAATGTAATGTG
36	CPS10-F	TCTACTGGGCAGACTGGTC
	CPS10-R	CGGTTTGTTTTGTCGTTTC
37	CPS11 -F	AACACGACGACCAATTCTTAC
	CPS11 -R	GCAGCTTGAGTAAAACGATC
38	CPS13 -F	TCCAGGCAGTAAATCAGACGC
	CPS13 -R	ATCAGCAGGTGTAGCCCCATC
39	CPS14-F	GGAGGAAGGGAATCAGTTCG
	CPS14-R	CGCAGACAACCAACTATCACG
40	CPS18-F	CACTAATCTTCCATCTTCTCC
	CPS18-R	TCTGTTTTGTGTTTGTTTGAC
41	CPS19-F	AAACCACTCACCTACTCACACTG

	CPS19-R	ATCAAGACCACCCATTCCAAG
42	CPS20-F	GAATGGAGAGCCCACTTGAC
	CPS20-R	ACATGGGGGTTTATAGAGGAG
43	CPS21-F	CATCTTGAACCCAAAACGAAC
	CPS21-R	GCCATCCTGAGTACCCGTG
44	CPS26-F	GTCTCTTTCTTTGTCCGCAC
	CPS26-R	AGGCGAAGTAGAGTTGAAGG
45	CPS27-F	GATGGGGAAAAGTAAGAAGT
	CPS27-R	GGTGGGGGATGTAAGTATGTA
46	CPS28-F	GCAACTCTCAACAACAAGGC
	CPS28-R	CAAAAGGAAATGATTGGGCT
47	CPS29-F	ACATTATTCGCTTCCCTTTC
	CPS29-R	GTCTCTTTCTTTGTCCGCAC
48	CPS30-F	CATCAAACAAACTATCCTCTCG
	CPS30-R	AGTATTTACCGTCACCAGCG
49	CPS31-F	GTCACCAGTCATCTTGCCAC
	CPS31-R	GTCTGTATTCCCAACAATCATC
50	CPS32-F	CAGCCAGAACCGTCTACATC
	CPS32-R	GCTCCATCACAAGTCCAACAT
51	C13690-2968F	TTTCAGACCGGACCTGATGC
	C13690-2968R	TCGACCATCGCATGACCAAA
52	C14034-1072F	TCGCTTTCACGTGGTAGTCC
	C14034-1072R	TGGAATGAGCGGAGGGAGTA
53	C16258_6223F	CTGTGGCCCATGACTCACTT
	C16258_6223R	CCTCCCTTCACGCAACATCT
54	C18580-17664F	GCTGTGGCTATTGTCCCTGT
	C18580-17664R	CCCGATATCCTCTTGTGGCG
55	C18610_15496F	AAAGCAGCTGAGAGACTGGG
	C18610_15496R	TCTCTCTGACTGGTAGGGGC
56	C18798_2744F	ACCCTTCCTCAGATCCTGCT
	C18798_2744R	GGGAGAGAAGACTTACCGGC
57	C19512_19723F	GTCGGCGTATCTTCCTCAGG
	C19512_19723R	ACGGCAGCCAATAAACGAGA
58	C19666_6151F	ACTTTCTGTCTCTCTCTTTTCCTCA
	C19666_6151R	GGAAGGTCGGACTCAACAGA
59	C19794-7345F	AGCCCAACACAGATGTCAGG
	C19794-7345R	GGACAGGACACATGACAGGG
60	C20054-10711F	TGGCTGTTGACCGTTCTTCA
	C20054-10711R	ACCTGCTGATCCGGGATAGT
61	C20508-14614F	ACGAAAATTTAAAGGATTCGCTCC
	C20508-14614R	GGAGTCCAAAATCCAGGATCCA

62	C20700-23477F	CAGAACTGAGAGGGTGCAGG
	C20700-23477R	TTTTGGATACTGGCGCTGGT
63	C21306-47773F	CGTGCTGGAGTCCCTGTTTA
	C21306-47773R	ACCGACCAAGACCAAAAGATCA
64	C21350-32182F	GAAAGATGGGGCAGGTGTGA
	C21350-32182R	GAAACCAGGAAGCCACCGTA
65	C21432-31744F	ACTTATGGCTTACCCGGTGC
	C21432-31744R	TGGATTCATTTGCCAGGAGC
66	C21464-37263F	GGCGGTGGATTGTCGAAAGA
	C21464-37263R	AGTAGGGAGAAATCAAAACACGA
67	C21474-46342F	GCCACAGGATCAGAGGAGGA
	C21474-46342R	GGCTGGGGTTTGGTTTAACG
68	C21604-13981F	TTCTTGCGGGGGTTTCACAT
	C21604-13981R	ACGTGTGTCAATTAGTGCCTG
69	C21636-15708F	CGTGTTTTCTCATGCGCACA
	C21636-15708R	TCCGTCGATCAATCTCGCTG
70	C21942-67901F	GTTGGTGTTGGTGTTGATCCA
	C21942-67901R	ATGAACAGGTCGGCTTAGGC
71	C22026-29919F	ATTCCTCTGCAGAAGACGCG
	C22026-29919R	GCCAATGCTCCATTTTCCCC
72	C22076-59411F	CGGGAAGTTCTGCACTTGGA
	C22076-59411R	ACCACAACTCACAAACCAAAAA
73	C22100-17348F	AAGTTCGAAGATCCCTCAA
	C22100-17348R	CAGGCTGGTTGAGACGACTA
74	C22100-279070F	ACCACTCTGCTGCTTCCATC
	C22100-279070R	GTCAAGCCGGACAGAGTTGA
75	C22152-57996F	ACCACCAACCCACCAACATT
	C22152-57996R	GGTCATCCTTGTTCAGGGGG
76	C22230-90014F	AAAGGGACAGGATCGTGGTG
	C22230-90014R	CCAACGCGAAACTTCCGTTT
77	C22298-60119F	CTCGGTCCATCAACACCCAA
	C22298-60119R	CTTGACTCTTCGGCTTCGGT
78	Scaffold140-292552F	ACGATGGAGGCTGCTATGTG
	Scaffold140-292552R	AAAACTGACGACGACGACGA
79	Scaffold141-159099F	GAGGTCAGCGACAAATTGGC
	Scaffold141-159099R	AAAGTTGGACCGGTTACGCT
80	Scaffold150-172691F	TGCTCCAGTCATGTACAGCTG
	Scaffold150-172691R	CAAGCCAGCTTACCCCAGAA
81	Scaffold172-35777F	TCAAGGAAGAACCGGGGAGA
	Scaffold172-35777R	CAAACACCGCTGGGACTTTG
82	Scaffold176-11303F	CTCCCCCTCAACACATGTCC

	Scaffold176-11303R	AGAGGGAGGAATACGAGTGAA
83	Scaffold179-435947F	TTCTGGACGGCATTTGCTTC
	Scaffold179-435947R	AGGAAGAGGCTAAGAGCGGT
84	Scaffold189-32318F	CGAGCACCAAAGGTTTAGCG
	Scaffold189-32318R	TGCATTAGCCACTCACCCAG
85	Scaffold20-114750F	TTCTTCTTCCTCGCCGAACC
	Scaffold20-114750R	GAAGAGGAGGGAGAGTGAGC
86	Scaffold20-115361F	CCCCCTCTCTCTTCCTTACCA
	Scaffold20-115361R	CTGCGCAACTGTATCACGTT
87	Scaffold20-27532F	GCGCAACTGTACGGCTTTAA
	Scaffold20-27532R	AGAACCAATAAAGTACTGTGAACGT
88	Scaffold203-77402F	AAGGAAGTGCGACAAGGTGT
	Scaffold203-77402R	TCCTACCCCTTCCCCTTCTG
89	Scaffold213-26650F	AGTGATGCAGCACTTCCAAA
	Scaffold213-26650R	CAGACATCGACGGGTGCATA
90	Scaffold217-99483F	TGTTGTTGTATGAACAGATGTTGT
	Scaffold217-99483R	ATCTGCACCTCAAATGGCCA
91	Scaffold221-17057F	TCGACCCTAACGGCATCCTA
	Scaffold221-17057R	TGATGCTGTTGAGAGCCAGG
92	Scaffold233-153774F	CCCTGTGCCTAAACTTCCCC
	Scaffold233-153774R	AGAGAAATCGAAGCGGTCAGA
93	Scaffold246-306327F	GCCAACAACAGCCTCGGTAT
	Scaffold246-306327R	ACCACTAATACTTCAAAACCTTCGA
94	Scaffold279-18233F	AGTTTGTGGAGGCGCATGTA
	Scaffold279-18233R	TGGCTGTTCTCCCTCCATTG
95	Scaffold284-32705F	TCGTGTCTCCTTCTCCTGGT
	Scaffold284-32705R	CGAGATCAAGAGTAGGCACGT
96	Scaffold476-71667F	CTGGCCAAAGCACTCACTCG
	Scaffold476-71667R	ATCAACCGGAAAGCGATCGA
97	Scaffold504-139032F	AACACAGACAGACATCTGAGTCA
	Scaffold504-139032R	ACTACACCTCCACCCATCCA
98	Scaffold510-69777F	GAGCGTTGGGTTTTGGGTTC
	Scaffold510-69777R	ACCATCCTCACTCTCGTCGA
99	Scaffold514-104058F	GAACCTGGAAACGGTTTGGC
	Scaffold514-104058R	CCACCCCCACTGTGTACAAA
100	Scaffold526-202442F	TCGTCTTCTGCTGATTCGACA
	Scaffold526-202442R	AGGAGACTCAGGATGGCAGA
101	Scaffold528-37649F	TCTCTCTCCTTCTCTCTTCTTCTCA
	Scaffold528-37649R	CCGAAAACCACACATGCCAG
102	Scaffold532-73542F	GCTGTGCGGAAAAGCGATAG
	Scaffold532-73542R	ACTCCGATCAGCACCCTACT

103	Scaffold600-19563F	AGGCGTGTTGAGTATTGAAGT
	Scaffold600-19563R	AGGCTCACAAAAGACCTGCT
104	Scaffold609-432549F	TGGGAAGCTGCCTTTATGCT
	Scaffold609-432549R	TCCACTCCCTTCCCATATCCT
105	Scaffold634-93796F	GCACCCACAGTTAAACAGACAC
	Scaffold634-93796R	ATCAGATCACCACCCCCGTA
106	Scaffold635-27016F	ATGCAACGGCGAAGTCAGTA
	Scaffold635-27016R	TGTCTCCTTGAGCTCCGTCT
107	Scaffold639-12819F	TCGTGAGGATTCAATTGGCCA
	Scaffold639-12819R	ATGCTGATGACCTTCTGGGC
108	Scaffold649-159654F	TGCAACAAAACTTGCCCAGG
	Scaffold649-159654R	TCGATAAACGCGGCGCTATA
109	Scaffold658-31701F	ACCGACAAAAACAAAAACGCA
	Scaffold658-31701R	CTCGGAAGGTTCACGCATCT
110	Scaffold662-164338F	CAACGCTCGATCGACTGGTA
	Scaffold662-164338R	CGAACGGGACAAGAAGGTGA
111	Scaffold672-45928F	TCGAAAGAAGCGAAGGAGGT
	Scaffold672-45928R	CAGCAGCCTCGATACCGAAT
112	Scaffold68-31780F	CCAGAGCAAGCAGGACTTCA
	Scaffold68-31780R	TCCTTCCCTCTCTCCACAGG
113	Scaffold689-22047F	GACCCACCTCACCTCAGAGT
	Scaffold689-22047R	CGCGGATCGGGACACTTAAA
114	Scaffold691-69709F	GATAGGAGCGGCTGAGCAAA
	Scaffold691-69709R	GGTACCGTTCTACTGGGTGG
115	Scaffold74-127705F	CGCAGTGTCACCTGAGTCTT
	Scaffold74-127705R	GGTACCTGGGTCCATTCAGC
116	Scaffold750-124306F	TTGGTTGTGGTTTGTCGGGA
	Scaffold750-124306R	GAGTCACTCTGCCGACCAAA
117	Scaffold750-152129F	AGTTGTTCGGGTGAGCTTGA
	Scaffold750-152129R	ACTAGCTCGAGTCATTGCCG
118	Scaffold774-64548F	TCCCAAGTCCCTAACCTGGT
	Scaffold774-64548R	CAGAGGGCACTGATCAGGAT
119	Scaffold782-387894F	CGCGAGCCCATGACTATCAA
	Scaffold782-387894R	GTGAGACCGTTCAGTTCAGGT
120	Scaffold793-76444F	AGCTCGGTTGGATGGATTGG
	Scaffold793-76444R	AGCGCGGTCAAGTCGATTAT
121	Scaffold806-86659F	CAGTAACCAACGCGGAGACT
	Scaffold806-86659R	AATCTGAAATCTCGCCCGCA
122	Scaffold810-81737F	AGAGGGGTGCATATGCCCTA
	Scaffold810-81737R	TCAAGTAGTTCTGCCACCAGT
123	Scaffold842-75625F	GTGGCTTCGGATCGATGGAT

	Scaffold842-75625R	ACGGTCTTGCTGCGTTAAGA
124	Scaffold843-63180F	GCACGCTTTCATGTACCACA
	Scaffold843-63180R	CATGTCTGTGACACTCGCCT
125	Scaffold851-201642F	TGAATGTGGAGGGTTGTGGA
	Scaffold851-201642R	AATTCAGAGCGAGTGGGCAA
126	Scaffold852-74142F	TTGACAAACTGGCGGATCCA
	Scaffold852-74142R	AGTGATACATCTCGATCCTCAACG
127	Scaffold853-116814F	TACCGGCTGTTCAGTTTGCT
	Scaffold853-116814R	ACTGGAAAGCAGTGCAAGGT
128	Scaffold90-11950F	CTAGTTCTCTGCCGGCTAGC
	Scaffold90-11950R	CCGTACCCCACACTGTCATC
129	Scaffold904-62635F	TTCTGGCGTGGGTTTGTCTT
	Scaffold904-62635R	ACCCTTCCTACCGTGACAGA
130	Scaffold912-43399F	ACGACTGTCCTCTGAGCTCA
	Scaffold912-43399R	CAACCGATTTTGGCCTGCAA
140	Scaffold913-279883F	CTCCACGATCTATGACGGCC
	Scaffold913-279883R	TGGCTTTACTCATAGCGTCGG
141	Scaffold960-65894F	TCTGCGCTATGGGATTCGAT
	Scaffold960-65894R	AGGTTTGATTGAGTCTGTGGTGT

Supplementary Table S3. List of 10 SSR primers for genotyping an isolate of a Pakistani dominant race (574232) of *Puccinia striiformis* f. sp. *tritici* (*PST*) and its 115 progeny isolates obtained from artificially inoculated *Berberis pseudumbellata*.

No.	SSR Primer	Sequences (5'–3')	Product Size Range (bp)	PIC
1	*Pst*P030–F	AAGGAAAAGAACTGTATG	304–315	0.305
	*Pst*P030–R	TTCAGATGCTCTATTCAA		
2	*Pst*P008–F	CCCTTGAGTAGTATGACC	454–457	0.218
	*Pst*P008–R	AGAAGAGGACGAGAAGAT		
3	SUNI*Pst*11–10 –F	CTGGTTTTGCTTTTAGGAGTTT	321–336	0.206
	SUNI*Pst*11–10 –R	AGGCAATGGTCCAGAAAAGAGT		
4	SUNI*Pst*10–06–F	GCGGCATTAGCGTCTCTTC	348–421	0.314
	SUNI*Pst*10–06–R	TCGCTTTCGTTCTCCATTGTC		
5	C21942–67901F	GTTGGTGTTGGTGTTGATCCA	279–307	0.316
	C21942–67901R	ATGAACAGGTCGGCTTAGGC		
6	C20054–10711F	TGGCTGTTGACCGTTCTTCA	230–250	0.325
	C20054–10711R	ACCTGCTGATCCGGGATAGT		
7	Scaffold189–32318F	CGAGCACCAAAGGTTTAGCG	281–293	0.227
	Scaffold189–32318R	TGCATTAGCCACTCACCCAG		
8	Scaffold912–43399F	ACGACTGTCCTCTGAGCTCA	208–226	0.214
	Scaffold912–43399R	CAACCGATTTTGGCCTGCAA		
9	Scaffold913–279883F	CTCCACGATCTATGACGGCC	258–272	0.328
	Scaffold913–279883R	TGGCTTTACTCATAGCGTCGG		
10	Scaffold514–104058F	GAACCTGGAAACGGTTTGGC	250–272	0.347
	Scaffold514–104058R	CCACCCCCACTGTGTACAAA		

Supplementary Table S4. Multi-locus genotypes (MLGs) of a Pakistani dominant race (574232) of *Puccinia striiformis* f. sp. *tritici* (*PST*) and its 115 progeny isolates determined based on 10 simple sequence repeat (SSR) markers.

MLG	No. of isolates	Marker 1		Marker 2		Marker 3		Marker 4		Marker 5		Marker 6		Marker 7		Marker 8		Marker 9		Marker 10		No. of homozygous loci
		PstP030		PstP008		SUNIPst11-10		SUNIPst10-06		C21942-67901		C20054-10711		Scaffold189-32318		Scaffold912-43399		Scaffold913-279883		Scaffold514-104058		
		a1	a2	a1	a2	a1	a2	a1	a2	a1	a2	a1	a2	a1	a2	a1	a2	a1	a2	a1	a2	
	Parent isolate	275	278	428	432	328	338	362	365	291	295	247	253	295	298	214	227	271	280	256	269	0
1	1	275	278	428	432	328	338	362	365	291	295	247	253	295	298	214	227	271	280	256	269	1
2	2	278	278	428	432	328	338	362	365	291	295	247	253	295	298	214	227	271	280	256	269	1
3	1	275	278	428	432	328	338	362	365	291	295	247	247	295	298	214	227	280	280	256	256	2
4	1	275	278	428	432	328	338	365	365	291	295	247	253	295	295	214	227	271	280	256	269	2
5	2	275	278	432	432	328	338	362	365	291	295	247	253	295	298	214	227	271	280	256	256	2
6	1	275	278	428	432	338	338	362	365	291	295	247	253	295	298	227	227	271	280	256	269	2
7	2	275	278	432	432	328	338	362	365	295	295	247	253	295	298	214	227	280	280	256	269	3
8	1	275	278	428	432	338	338	362	365	291	295	253	253	295	298	214	227	280	280	256	269	3
9	2	278	278	428	432	328	328	365	365	291	295	253	253	295	298	214	214	280	280	256	256	3
10	1	275	278	428	428	328	338	362	365	291	291	247	253	298	298	214	227	271	280	256	269	3
11	1	275	278	428	432	338	338	362	365	291	295	247	247	295	298	214	227	271	280	256	256	3
12	1	278	278	428	432	328	338	365	365	291	295	247	247	295	298	227	227	271	280	256	269	4
13	1	275	278	428	428	338	338	362	362	295	295	247	253	295	298	214	227	271	280	256	269	4
14	1	275	278	432	432	328	338	362	365	291	295	253	253	298	298	214	227	271	271	256	269	4
15	1	275	278	428	432	328	338	362	362	291	295	253	253	295	298	214	214	271	280	269	269	4
16	1	275	275	428	432	338	338	365	365	295	295	247	253	295	298	214	227	271	280	256	269	4
17	1	275	278	428	428	328	338	365	365	291	295	247	247	295	295	214	227	271	280	256	269	4
18	1	275	278	428	432	338	338	362	365	291	291	247	253	295	298	227	227	271	271	256	269	4
19	1	278	278	428	432	328	338	365	365	291	295	253	253	295	298	214	227	271	280	269	269	4
20	1	275	278	428	428	338	338	362	362	291	295	253	253	298	298	214	227	271	280	256	269	4
21	2	275	278	428	432	328	338	362	362	291	295	247	253	298	298	227	227	271	271	256	269	4
22	1	275	278	432	432	338	338	362	365	291	295	247	253	295	298	214	227	280	280	256	256	4
23	1	278	278	428	432	328	338	365	365	295	295	253	253	295	298	227	227	271	280	256	269	5
24	1	275	278	428	432	338	338	362	365	295	295	247	253	298	298	214	214	271	280	256	256	5
25	1	275	275	428	432	328	338	362	362	291	295	N/A	N/A	298	298	214	227	271	271	256	269	5
26	3	275	278	428	428	338	338	362	365	291	295	247	247	295	298	227	227	271	280	256	256	5
27	4	275	278	428	432	328	338	362	365	295	295	247	247	298	298	214	227	280	280	256	269	5
28	2	278	278	432	432	328	338	362	362	291	295	247	247	295	298	227	227	271	280	256	269	5
29	2	275	278	428	428	328	338	362	365	291	291	247	253	298	298	227	227	280	280	269	269	5
30	2	278	278	428	432	328	328	362	365	295	295	247	253	298	298	214	214	271	280	256	269	5
31	2	275	278	428	432	328	338	365	365	291	291	247	247	295	298	214	214	271	271	256	269	5
32	2	275	278	428	432	328	338	362	365	291	295	247	247	298	298	227	227	271	271	269	269	5
33	2	275	275	428	432	338	338	362	362	291	295	253	253	295	298	214	227	271	280	269	269	5
34	2	275	278	432	432	328	338	362	365	295	295	253	253	295	298	214	214	280	280	256	269	5
35	2	278	278	432	432	338	338	362	365	295	295	247	253	295	298	214	227	271	280	256	256	5
36	2	275	278	432	432	328	338	365	365	291	291	247	253	295	298	227	227	280	280	256	269	5
37	2	278	278	428	432	338	338	362	365	291	295	247	247	298	298	214	227	271	271	256	269	5
38	2	275	278	428	428	328	338	362	362	291	295	247	247	295	295	227	227	271	280	256	269	5
39	2	278	278	428	428	328	338	365	365	291	291	247	247	295	295	214	227	271	280	256	269	6
40	3	275	275	428	432	338	338	362	365	291	291	253	253	298	298	214	227	280	280	256	256	6
41	3	275	275	428	428	328	338	362	362	291	295	253	253	295	298	214	214	271	280	256	256	6
42	3	278	278	432	432	328	328	362	365	295	295	247	253	295	298	214	214	271	271	256	269	6
43	3	275	278	432	432	328	338	365	365	295	295	253	253	295	298	227	227	271	280	269	269	6
44	3	278	278	428	432	338	338	365	365	295	295	247	253	298	298	214	227	271	280	269	269	6
45	4	275	278	432	432	338	338	365	365	295	295	253	253	295	298	214	214	271	280	256	269	6
46	4	278	278	428	428	328	338	365	365	291	295	253	253	295	295	214	227	280	280	256	269	6
47	4	278	278	428	432	338	338	362	362	295	295	247	253	295	295	227	227	271	280	256	269	6
48	4	278	278	432	432	328	328	362	362	291	295	247	253	295	295	227	227	271	280	256	256	7

49	4	275	278	428	432	328	328	362	362	295	295	247	247	298	298	227	227	280	280	256	256	7
50	4	275	278	432	432	338	338	362	362	291	291	253	253	295	298	214	227	280	280	269	269	7
51	3	278	278	428	432	328	328	365	365	295	295	253	253	295	298	214	214	271	280	269	269	7
52	2	278	278	428	428	338	338	365	365	295	295	247	253	298	298	214	227	271	280	256	256	7
53	1	275	275	428	432	338	338	362	365	295	295	247	253	298	298	227	227	280	280	256	256	7
54	1	275	275	428	432	338	338	362	362	295	295	247	247	298	298	214	214	271	271	256	269	8
55	2	275	275	432	432	328	328	362	362	291	291	247	247	295	295	227	227	271	280	256	256	8
56	1	275	278	428	428	338	338	365	365	295	295	253	253	295	298	227	227	271	271	269	269	8
57	1	275	275	432	432	328	328	362	362	291	295	247	253	295	295	214	214	280	280	269	269	8
58	2	275	278	432	432	328	328	365	365	291	291	253	253	295	295	227	227	280	280	256	269	8
59	1	275	278	432	432	328	328	365	365	295	295	253	253	298	298	214	214	280	280	269	269	9
60	1	278	278	428	428	328	338	362	362	291	291	247	247	298	298	214	214	271	271	269	269	9

The 10 SSR markers used are: **1** = *Pst*P030, **2** = *Pst*P008, **3** = SUNI*Pst*11–10, **4** = SUNI*Pst*10–06, **5** = C21942–67901, **6** = C20054–10711, **7** = Scaffold189–32318, **8** = Scaffold912–43399, **9** = Scaffold913–279883, **10** = Scaffold514–104058. Sizes of amplified fragments are given in base pairs. a1 = allele 1, and a2 = allele 2. MLG = multi–locus genotype. NB = no band.
